# Digital Clock and Recall: a digital, process-driven evolution of the Mini-Cog

**DOI:** 10.3389/fnhum.2024.1337851

**Published:** 2024-08-26

**Authors:** Joyce Gomes-Osman, Soo Borson, Claudio Toro-Serey, Russell Banks, Marissa Ciesla, Ali Jannati, W. Isaiah Morrow, Rod Swenson, David Libon, David Bates, John Showalter, Sean Tobyne, Alvaro Pascual-Leone

**Affiliations:** ^1^Linus Health, Inc., Boston, MA, United States; ^2^Department of Neurology, University of Miami Miller School of Medicine, Miami, FL, United States; ^3^Department of Family Medicine, Keck School of Medicine, University of Southern California, Los Angeles, CA, United States; ^4^Department of Psychiatry and Behavioral Sciences, University of Washington, Seattle, WA, United States; ^5^Department of Communicative Sciences and Disorders, College of Arts and Sciences, Michigan State University, East Lansing, MI, United States; ^6^Department of Neurology, Harvard Medical School, Boston, MA, United States; ^7^Department of Psychiatry and Behavioral Science, University of North Dakota School of Medicine and Health Sciences, Fargo, ND, United States; ^8^Department of Geriatrics and Gerontology, New Jersey Institute for Successful Aging, Rowan University School of Osteopathic Medicine, Stratford, NJ, United States; ^9^Hinda and Arthur Marcus Institute for Aging Research and Deanna and Sidney Wolk Center for Memory Health, Hebrew SeniorLife, Boston, MA, United States

**Keywords:** Digital Clock and Recall, Mini-Cog, dementia, cognitive screen, next-generation, process-driven

## Abstract

**Introduction:**

Alzheimer’s disease and related dementias (ADRD) represent a substantial global public health challenge with multifaceted impacts on individuals, families, and healthcare systems. Brief cognitive screening tools such as the Mini-Cog© can help improve recognition of ADRD in clinical practice, but widespread adoption continues to lag. We compared the Digital Clock and Recall (DCR), a next-generation process-driven adaptation of the Mini-Cog, with the original paper-and-pencil version in a well-characterized clinical trial sample.

**Methods:**

DCR was administered to 828 participants in the Bio-Hermes-001 clinical trial (age median ± SD = 72 ± 6.7, IQR = 11; 58% female) independently classified as cognitively unimpaired (*n* = 364) or as having mild cognitive impairment (MCI, *n* = 274) or dementia likely due to AD (DLAD, *n* = 190). MCI and DLAD cohorts were combined into a single impaired group for analysis. Two experienced neuropsychologists rated verbal recall accuracy and digitally drawn clocks using the original Mini-Cog scoring rules. Inter-rater reliability of Mini-Cog scores was computed for a subset of the data (*n* = 508) and concordance between Mini-Cog rule-based and DCR scoring was calculated.

**Results:**

Inter-rater reliability of Mini-Cog scoring was good to excellent, but Rater 2’s scores were significantly higher than Rater 1’s due to variation in clock scores (*p* < 0.0001). Mini-Cog and DCR scores were significantly correlated (*τ*_B_ = 0.71, *p* < 0.0001). However, using a Mini-Cog cut score of 4, the DCR identified more cases of cognitive impairment (*n* = 47; *χ*^2^ = 13.26, *p* < 0.0005) and Mini-Cog missed significantly more cases of cognitive impairment (*n* = 87). In addition, the DCR correctly classified significantly more cognitively impaired cases missed by the Mini-Cog (*n* = 44) than vice versa (*n* = 4; *χ*^2^ = 21.69, *p* < 0.0001).

**Discussion:**

Our findings demonstrate higher sensitivity of the DCR, an automated, process-driven, and process-based digital adaptation of the Mini-Cog. Digital metrics capture clock drawing dynamics and increase detection of diagnosed cognitive impairment in a clinical trial cohort of older individuals.

## Background

The number of individuals living with Alzheimer’s disease and related dementias (ADRD) will more than double over the next 25 years, reaching 13 million people in the United States at an estimated nearly $1 trillion dollars of direct healthcare costs (not including unpaid care) (Alzheimer’s Association, 2023). Over the next 10 years, it is anticipated that 1.2 million new healthcare workers will be needed to provide care and services for individuals living with dementia, the largest workforce shortage in the US (Alzheimer’s Association, 2021). This problem is further complicated by the growing shortage of medical specialists prepared to address the panoply of issues associated with dementia, especially neurologists (Dall et al., 2013; Majersik et al., 2021). Moreover, widespread gaps remain in the detection and diagnosis of cognitive disorders, including mild cognitive impairment (MCI) and dementia (Mattke et al., 2023). This challenge is particularly critical in sociodemographically disadvantaged populations. Projections suggest that by 2025 about 70% cases of dementia worldwide will come from these populations, making the need for reliable and scalable screening tools particularly critical (Nichols et al., 2022).

Primary care providers (PCP) are well-positioned to screen and detect for emerging cognitive impairment. Though they are among the most time-pressured professionals in healthcare (Bransby et al., 2019), many consider that dementia detection is well within their scope of practice but would improve with more training (Sideman et al., 2023). In another survey, only 40% of PCPs were familiar with existing cognitive screening tools (Alzheimer’s Association, 2021). Though biomarkers are an emerging standard for defining Alzheimer’s disease, including in presymptomatic stages (Alzheimer’s Association, 2024), cognitive decline remains a critical hallmark feature of dementia, with the progression of dementia responsible for its most devastating impact on patients and their families (Nichols et al., 2022). Clinicians need cognitive tests to identify individuals who are experiencing cognitive decline at early symptomatic stages to enable timely interventions.

Among individuals whose cognitive impairment is due to Alzheimer’s disease (AD), minimizing the progression of cognitive decline is the primary goal of amyloid-targeting therapies, and therapeutic response is most likely—and largest—for individuals receiving treatment early, when cognitive deficits are mild (McDade et al., 2022; Brum et al., 2023). However, for the estimated 70% of people with mild cognitive impairment (MCI) or more advanced cognitive impairment who do not qualify for anti-amyloid therapies (Pittock et al., 2023), objective assessments of cognitive performance are important to guide clinical and lifestyle management interventions. Promoting a sense of agency, early interventions can improve cognitive outcomes as well as promote longevity, quality of life, and functional independence (Kivipelto et al., 2018; Livingston et al., 2020).

Cognitive screening tools such as the Mini-Cog© are widely used in primary care (Borson et al., 2005; Kamenski et al., 2009). Initially conceptualized as a “cognitive vital sign,” the Mini-Cog combines a clock drawing task with short-term verbal recall (Borson et al., 2005) using empirically derived, plain-language scoring rules. With a low training burden, the Mini-Cog allows for quick and easy administration while providing a low-cost option for non-specialist healthcare professionals to streamline the detection of cognitive impairment under the supervision of a physician or a psychologist. The Mini-Cog has been used in multiple studies in healthcare and community-based settings, including the Medicare Annual Wellness Visit—the only Medicare benefit to require detection of cognitive impairment as a condition of reimbursement.

We developed a process-driven, next-generation version of the Mini-Cog, Digital Clock and Recall (DCR™), which captures the dynamic process of drawing a clock. These metrics provide important information beyond the simple accuracy scores for recall and clock drawing tests used in the original Mini-Cog. Whereas the Mini-Cog and other paper based cognitive assessments evaluate the final drawn image, the process-based analysis afforded with the DCR accounts for drawing efficiency (e.g., drawing size and stroke count), visuospatial awareness (e.g., number and hand placement), simple and complex motor (e.g., drawing speed and consistency), and information processing (e.g., time and latency variables), all of which are components of neurodegenerative processes that may be difficult to observe in face-to-face encounters. The DCR leverages a digital platform utilizing machine learning to analyze latency and graphomotor behaviors from clock drawing and acoustic features extracted from verbal recall (Banks et al., 2022; Hammers et al., 2023; Jannati et al., 2023a). Process-driven automatic scoring of the clock drawing test conditions (command and copy) are associated with early signs of β-amyloid burden in the brain, enabling the detection of clinically meaningful AD pathology at early (“presymptomatic”) stages, at least in highly selected research participants (Rentz et al., 2021). The application of similar approaches to delayed verbal recall has further improved its predictive accuracy for classifying AD-related brain pathology such as β-amyloid and tau (Bates et al., 2023).

The goal of the present study was to compare (1) the process-driven automatic scoring of the DCR and (2) blinded *a priori* visual scoring of the same (digitally drawn) clocks and delayed recall from two experienced clinical neuropsychologists using the original Mini-Cog scoring rules. We focused specifically comparing identification of positive (cognitively impaired) cases in this analysis. To answer this question, we conducted a retrospective analysis of previously collected data from 945 participants identified *a priori* as neurologically healthy, having mild cognitive impairment, or probable AD dementia based on clinical diagnosis verified by electronic medical records or performance using a protocol of neuropsychological and functional-assessment tests including the Mini-Mental State Examination (MMSE), the Rey Auditory Verbal Learning Test (RAVLT), and the informant-rated Functional Activity Questionnaire (FAQ). We assessed the interrater reliability of clocks hand-scored using the Mini-Cog criteria by two highly experienced neuropsychologists (each with 30+ years of practice), and the criterion-related validity of hand- vs. DCR-scored clocks and total score for 3-item recall and clock drawing against the pre-specified diagnostic classification.

## Methods

### Sample

An original sample of 945 older adults (57% female, aged 72 ± 6.7 yrs.) completed a battery of cognitive and motor function tests, including the DCR, as part of the Bio-Hermes-001 study on brain health (ClinicalTrials.gov ID: NCT04733989) (see Table 1 for demographic information). The multi-site, multi-visit Bio-Hermes-001 study, managed by the Global Alzheimer’s Platform (GAP), investigated state-of-the-art methods in brain health research (Beauregard et al., 2022). The study used blood, positron emission tomography (PET), and digital biomarkers in a large, racially diverse sample of older adults to yield the three distinct cohorts included in the present study (see below): healthy (no cognitive impairment), mild cognitive impairment (MCI), and dementia likely due to AD (DLAD). Ethical approval was granted by the Institutional Review Board at each institution participating in the GAP consortium. All participants provided written informed consent to participate in the study. Inclusion criteria were adults 60–85 years of age, fluent in the language of the tests used at the site, and with a MMSE score of 20–30 at screening. Exclusion criteria were extensive and based on underlying conditions that could confound interpretation of results; more information can be obtained through the GAP consortium^1^ (Mohs et al., 2024).

**Table 1:** Demographic information for each of the resulting cohorts.

	Cognitively unimpaired % or Median (SD, IQR)	Cognitively impaired % or Median (SD, IQR)	Comparison test
Total *N*	364	464	
Sex (Females)	61%	55%	*χ*^2^ = 2.26, *p* = 0.13
Ethnicity (Hispanic)	6%	10%	*χ*^2^ = 4.50, *p* < 0.05
Race (White)	86%	84%	*χ*^2^ = 57.94, *p* = 0.44
Age in years	70 (6.4, 10)	74 (6.6, 10)	*T* = −5.96, *p* < 0.0001
Years of education	16 (2.4, 4)	16 (2.8, 4)	*W* = 94,566, *p* < 0.005
MMSE	29 (1.4, 2)	26 (2.8, 4)	*W* = 134,465, *p* < 0.0001
RAVLT long delay	9 (2.5, 3)	4 (2.6, 4)	*W* = 150,393, *p* < 0.0001
PET Aβ positivity	22%	43%	*χ*^2^ = 37.49, *p* < 0.001

Total *N* was 828. For DCR vs. Mini-Cog scoring analysis, we only used the first engagement of a participant with the DCR. *χ*^2^ refers to a chi-squared test for equality of proportions. The *T*-values provided are the statistics for independent-samples *T*-tests. *W*-value refers to the statistic for Wilcoxon rank sum tests. *χ*^2^ refers to a chi-squared test for equality of proportions. The *T*-values provided are the statistics for independent-samples *T*-tests. *W*-value refers to the statistic for Wilcoxon rank sum tests. MMSE scores >25 are considered cognitively normal, while <24 indicate cognitive impairment due to dementia (Creavin et al., 2016). The RAVLT Long Delay score is performed following a 30-min delay and is scored according to published age-adjusted norms (Mitrushina et al., 2005).

The original sample of 945 participants was subsampled twice, once for each goal of the study. The first sample, configured for assessing inter-rater reliability, contained 508 participants evaluated by two neuropsychologists (total unimpaired = 251, total MCI = 173, total DLAD = 84; age median ± SD = 72 ± 6.7, IQR = 11; 49% female; years of education median ± SD = 16 ± 2.5, IQR = 4; 87.4% White; 9.2% Black or African–American; 1.7% Asian; 7.4% Hispanic or Latino). The smaller size was due to Rater 1 having scored tests from fewer participants than Rater 2. The second subsample contained 828 participants (age median ± SD = 72 ± 6.7, IQR = 11; 58% female; years of education median ± SD = 16 ± 2.6, IQR = 5; primary language English; 85.6% White; 11.8% Black or African–American; 1.4% Asian; 8.8% Hispanic or Latino), which included all the participants scored by Rater 2. This larger sample was used for evaluating differences in scoring between tests, and only the first test per participant was evaluated.

### Cohort designation

All 828 participants were classified *a priori* by the Bio-Hermes study team into cognitively unimpaired (CU; *n* = 364), mild cognitive impairment (MCI; *n* = 274), or probable AD dementia (DLAD; *n* = 190). Cohort classification criteria included the MMSE score and RAVLT Long Delay Recall Score. Therefore, we refer to these groups as CU, and cognitively impaired (CI; made up of MCI and DLAD cohorts). See Table 1 for demographic information per cohort.

### DCR administration

The DCR, a brief digital cognitive assessment composed of Immediate Recall, clock drawing (DCTclock™), and Delayed Recall, was administered via an iDLAD Pro (11″, 4th Generation, Apple, CA, USA) with a stylus (Apple Pencil). The Immediate and Delayed Recall components of the DCR consist of three words. Individuals are asked to repeat these words immediately (Immediate Recall), complete the DCTclock, and then recall the three words after a delay (Delayed Recall). Delayed Recall assesses verbal episodic memory, which is particularly impaired at the early stages of AD (Leube et al., 2008; Wolk et al., 2011; Gallagher and Koh, 2011). Evaluation of verbal episodic memory is important for classifying the patient’s current cognitive status and for estimating the likelihood of the patient’s progression to dementia over the subsequent decade (Silva et al., 2012; Belleville et al., 2017). The DCTclock is composed of a Command Clock condition (as in the original Mini-Cog) followed by a Copy Clock condition (not part of the original Mini-Cog). In the Command Clock condition, the task is to draw an analog clock from memory with hands set to “10 after 11,” whereas the Copy Clock condition involves copying an already-drawn template clock set to the same time. Participants take the DCR only once per visit.

### Scoring of Digital Clock and Recall

There are three steps to scoring the DCR: (1) automatic transcription and scoring of delayed recall; (2) automated scoring of clock drawing performance; and (3) combination of recall and clock scores. For the first score, there is no time limit for the Immediate and Delayed Recall tasks. Each word recalled correctly in the Delayed Recall contributes one point (for a maximum of three points) toward the total DCR score. Immediate Recall does not directly contribute to the overall DCR score. The DCR application records the patient’s voice response following the three-word prompt separately for the Immediate and Delayed Recall segments. These recordings are converted to text through an automated speech recognition (ASR) algorithm and then compared against the originally presented words to calculate the Delayed Recall accuracy. These recordings can also be analyzed for acoustic features, in what we define as DCR plus, which has been shown to be superior to the DCR in detecting cognitive impairment (Banks et al., 2022). Internal validation of the ASR algorithm compared to a human transcriber has shown a 5%-word error rate.

In terms of the second score, the DCTclock contributes up to two points to the overall DCR score. The design and implementation of the DCTclock data analysis engine have been previously reported in detail (Mattke et al., 2023; Souillard-Mandar et al., 2016). Briefly, the measures that are derived from the DCTclock are summarized in a single summary score out of 100 with cutoff scores of <60, 60–74, and ≥75, contributing 0, 1, and 2 points to the total DCR score, respectively. The DCTclock includes four Command and Copy Clock composite scales, each composed of 22 subscales that evaluate various aspects of the clock-drawing process: drawing efficiency, information processing, simple and complex motor skills, and spatial reasoning (Rentz et al., 2021; Souillard-Mandar et al., 2016; Matusz et al., 2022). Out of 1891 DCR tests performed and automatically scored across visits in the original data set, only 4% of DCTclocks were deemed insufficiently completed by the participant and required manual scoring. More than half of those were not from a participant’s first test, the test evaluated here. Therefore, no incomplete clocks were included in the present analyses.

Finally, the total DCR score is a combination of the DCTclock and the Delayed Recall scores and is presented as a total score of 0–5. The DCTclock and the Delayed Recall contribute 0–2 points and 0–3 points to the DCR score, respectively. A DCR score of 4–5 means that no indication of cognitive impairment was detected. Individuals with a 2–3 DCR score are considered borderline for cognitive impairment. Individuals with a 0–1 DCR score are considered likely to have cognitive impairment.

### Mini-Cog scoring

DCR clocks were scored using Mini-Cog criteria by two experienced clinical neuropsychologists. Unlike the DCTclock protocol, the Mini-Cog allows the option of beginning with a pre-drawn circle representing the clock face. For this reason, the circular clock face is not accounted for in the Mini-Cog scoring as it is in most other manual clock-scoring schemes and the DCTclock’s automated scoring algorithms. The Mini-Cog scoring guidelines^2^ are as follows:

Recall Score (Total Possible Score: 0–3)

- 1 point for each word correctly recalled without prompt

Clock Drawing Score (Possible Scores: 2 or 0)

- 2 points for a normal clock
  - A normal clock must include all numbers (1–12), each only once, in the correct order and direction (clockwise).
  - There must also be two hands present, one pointing to the 11 and one pointing to 2.
- 0 (zero) points for an abnormal clock drawing

### Analyses

#### Inter-rater reliability

Two independent clinical neuropsychologists (Raters 1 and 2) with a combined 70+ years of experience (35+ years each) administering neuropsychological tests including the Mini-Cog and blinded to participants’ cognitive cohort, rated recall and the final image of digitally drawn clocks using traditional Mini-Cog scoring guidelines. The neuropsychologist ratings were used in both phases of our analysis. In a multi-ethnic, multi-lingual sample with varying education that was used for initial validation of the Mini-Cog, a total score of 0, 1, or 2 was found to best discriminate higher from lower likelihood of clinically important cognitive impairment. A total Mini-Cog score of 3, 4, or 5 indicated a lower likelihood of dementia but did not rule out some degree of cognitive impairment (Borson et al., 2005).

A common set of 508 participants was used to evaluate inter-rater reliability (see Sample section for demographic details). We took two approaches to assessing inter-rater agreement: intraclass correlations (ICC) and paired permutations comparing median scores. For ICCs, we used the ICC (3,1) formulation given by Shrout and Fleiss (1979), using the Psych package for *R* based on linear mixed-effects models (Revelle, 2023). This formulation is not meant to generalize agreement to a population of Raters, which has been the aim of other studies (Scanlan and Borson, 2001; Borson et al., 2005), and does not explicitly account for differences in mean ratings. To examine differences in rating, we performed paired permutations for each metric (e.g., separately for recall and clock scores). On each of the 5,000 iterations, scores from the given metric were randomly reassigned to each Rater, and the difference in mean scores was computed. The resulting 5,000 differences were then used as a null distribution to calculate the probability that a score could have been at least as extreme as the empirical difference between Raters (i.e., the *p*-value). Alpha for all statistical tests was set to 0.05.

#### Overall score thresholding comparison of cognitive impairment classification

Since both of our expert Raters scored both Copy and Command clocks, we compared the DCR score against the clock version that had the highest inter-rater reliability. Further, since each Rater scored a slightly different sub-sample of the original BioHermes data set, we chose to use ratings from the Rater whose Visit 1 sub-sample had the highest number of participants (this resulted in our sample of 828 clocks; see Table 1 for demographic information).

We first compared summary scores between automated DCR and Rater scoring. First, we evaluated the level of agreement in recall scores between DCR and the selected Rater. We did so via ICC and paired permutations, as done previously between Raters (see *Inter-Rater Reliability* for details). In this way, we checked whether differences between overall test scores of DCR and Mini-Cog were due simply to the clock evaluation or recall as well. We then computed the Kendall’s Tau (*τ*_B_) rank correlation between Mini-Cog and DCR scores. Next, we evaluated the sensitivity of each scoring system by computing the number of impaired individuals that were missed by each test under a score cutoff of 4 (i.e., individuals who scored 4 or 5 under either test’s rules were deemed to be cognitively unimpaired). This cutoff was chosen because (1) it is the designed cutoff for distinguishing between cognitively impaired and unimpaired by the DCR (see Scoring of Digital Clock and Recall for details); and (2) it was meant to increase the sensitivity of Mini-Cog to mild cognitive impairment, rather than to dementia only (Borson et al., 2000; Borson et al., 2003). We complemented this analysis by tallying up the number of such missed cases that were correctly captured by the opposite scoring approach. We statistically evaluated these comparisons using a chi-squared (*χ*^2^) test of proportions with continuity correction. Finally, we calculated key classification metrics (i.e., sensitivity, specificity, negative predictive value [NPV], and positive predictive value [PPV]) for each score threshold of the DCR and Mini-Cog. We employed bootstrap procedures to estimate 95% confidence intervals (CI) for each of these metrics, as well as to statistically compare the difference of these metrics between tests. This analysis worked as follows: for each threshold, we resampled target positive (impaired) and target negative (unimpaired) groups separately and with replacement 5,000 times, ensuring that the proportion of each group resembled that of the original data set. We then calculated the aforementioned classification metrics based on the predefined cohorts. The 95% CI was calculated based on the percentiles of the bootstrap distribution of each metric. A statistically significant difference was defined by taking the difference between the 5,000 bootstrap values per metric between tests and checking if the 95% CI of this difference did not include 0.

## Results

### Sample

We studied 828 participants from a multisite, multi-visit study (age median ± SD = 72 ± 6.7, IQR = 11; 58% female; years of education median ± SD = 16 ± 2.6, IQR = 5; primary language English; 85.6% White; 11.8% Black or African–American; 1.4% Asian; 8.8% Hispanic or Latino), classified *a priori* as cognitively unimpaired (*n* = 364), ‘MCI’ (*n* = 274), or ‘DLAD dementia’ (*n* = 190). MCI and DLAD cohorts were combined into a single CI cohort for all comparisons. This sample comprises all participants from an original sample of 945 who were scored by Rater 2 (see Methods for details). Table 1 shows these demographic characteristics stratified by cohort, as well as percentage of PET Aβ positivity. A subsample of 508 of these 828 were used for inter-rater reliability (Total unimpaired = 251, total MCI = 173, total DLAD = 84; age median ± SD = 72 ± 6.7, IQR = 11; 49% female; years of education median ± SD = 16 ± 2.5, IQR = 4; 87.4% White; 9.2% Black or African–American; 1.7% Asian; 7.4% Hispanic or Latino). This smaller sample is due to Rater 1 scoring fewer participants than Rater 2.

### Inter-rater reliability of Mini-Cog scoring

The inter-rater reliability analysis showed that most ICC (3,1) values ranged from good to excellent, except for the reduced agreement (Koo and Li, 2016) of Copy Clock scores (Table 2) which is not part of the original Mini-Cog. Paired permutation comparisons indicated that Rater 2 scored significantly higher than Rater 1 across all conditions (*p* < 0.0001, Bonferroni-corrected) except for delayed recall (*p* > 0.05).

**Table 2:** Results from inter-rater reliability analyses based on 547 participants.

Score	Rater 1 Mean (SD)	Rater 2 Mean (SD)	% Agreement (between raters)	Intraclass correlation (ICC 3,1)	95% Confidence interval	ICC *p*-value
Recall score (0–3)	1.31 (1.15)	1.35 (1.14)	93.2	0.94	0.93–0.95	<0.0001
Copy clock rating (0 or 2)	1.75 (0.65)	1.92 (0.37)	91.2	0.41	0.34–0.48	<0.0001
Command clock rating (0 or 2)	1.35 (0.93)	1.51 (0.85)	89.5	0.76	0.72–0.80	<0.0001
Copy-based total Mini-Cog score (0–5)	3.07 (1.37)	3.27 (1.23)	85.4	0.87	0.85–0.89	<0.0001
Command-based total Mini-Cog score (0–5)	3.66 (1.64)	2.87 (1.60)	84.1	0.88	0.86–0.90	<0.0001

Confidence intervals are two-sided at 95%. Copy Clock- and Command Clock-based scores refer to Mini-Cog scoring rules applied to each of these DCR conditions. ‘*% Agreement*’ refers to the percentage of times the Raters produced identical scores. ICC values are the result of a comparison between Rater 2 Mini-Cog scoring and automated DCR scoring.

### Relationship between Mini-Cog and DCR scoring

**Figure 1:**
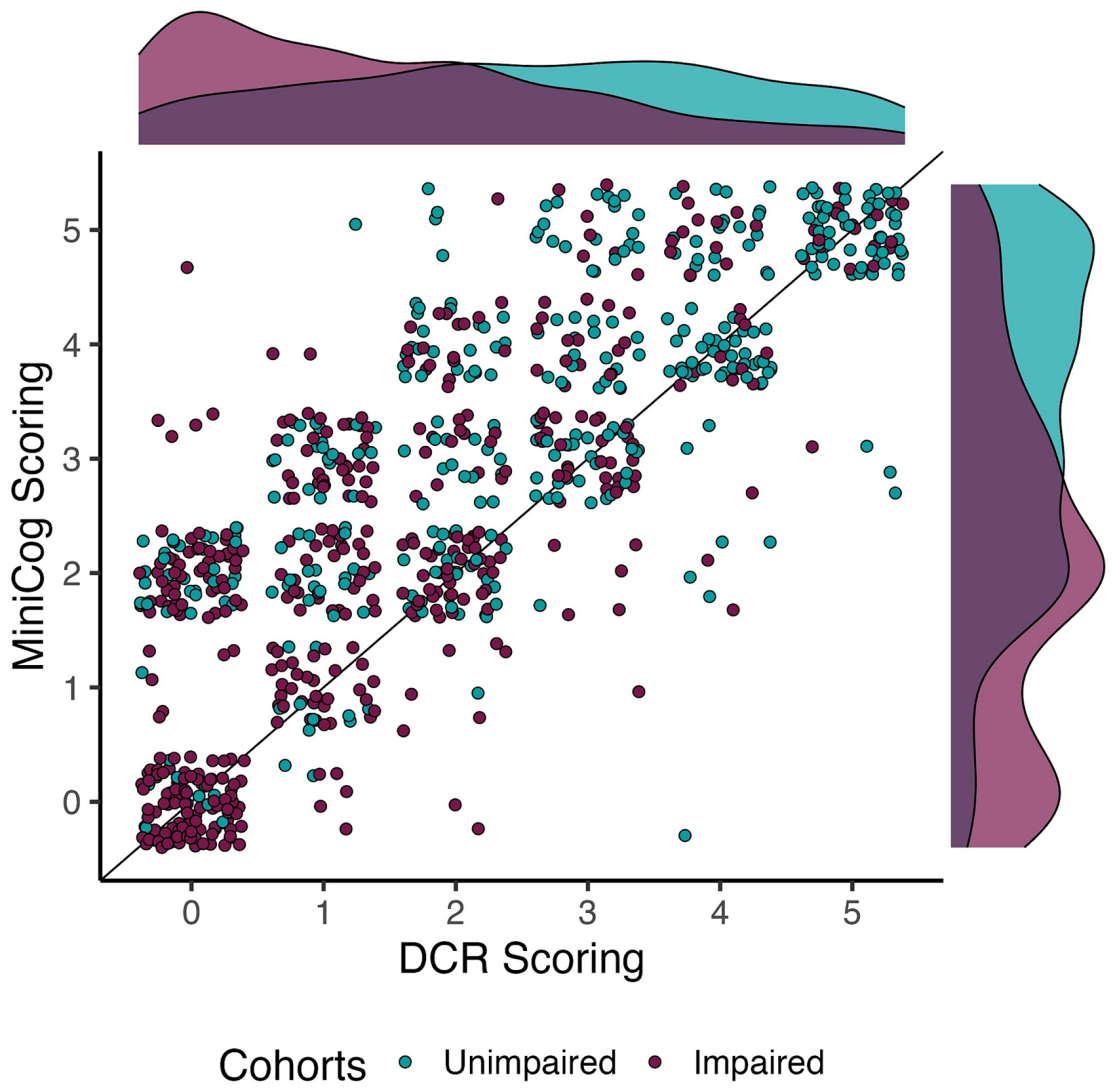
Relationship between DCR and Mini-Cog scoring per cohort. Dots are jittered to facilitate visualization of score prevalence. The apparent correlation between scores was confirmed by a significant and moderate Kendall’s Tau correlation (*τ*_B_ = 0.71, *p* < 0.0001). Note that DCR tended to score impaired individuals quite low relative to the Mini-Cog.

Given the high agreement between Raters on the Command Clock scoring, and the higher number of unique participants scored by Rater 2, Rater 2’s scores were used to assess the relationship and comparison between DCR and Mini-Cog scoring. We note that results do not meaningfully change when using Rater 1 instead. Figure 1 illustrates the distribution of scores across individuals classified as CU and CI. While analyses demonstrated a significant correlation between the two assessments (Kendall’s *τ*_B_ = 0.71, *p* < 0.0001), we found that mean DCR scores were significantly lower than the Mini-Cog rubric (paired permutation *p* < 0.0001). However, this was not due to mean differences in delayed recall scores (paired permutation *p* > 0.05), suggesting that differences in sensitivity were likely due to clock-scoring rules.

### Higher sensitivity of DCR scoring

**Figure 2:**
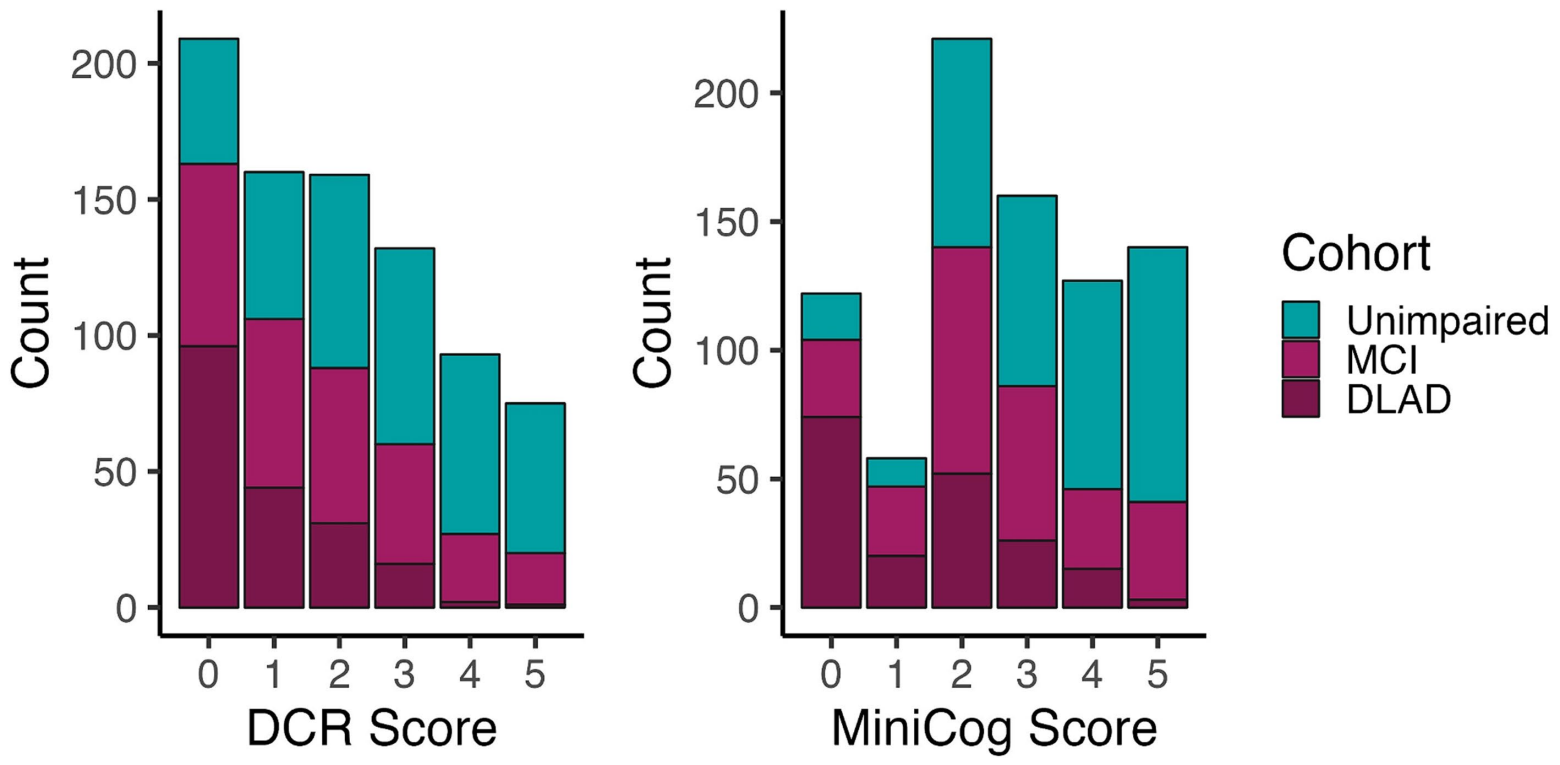
Number of individuals in each group for each test’s score. The distribution shows that the DCR produced more false positives (*n* = 171) than Mini-Cog (*n* = 110) at a score threshold of 4, but DCR captured a greater proportion with CI (*n* = 357) than the Mini-Cog (*n* = 291).

**Figure 3:**
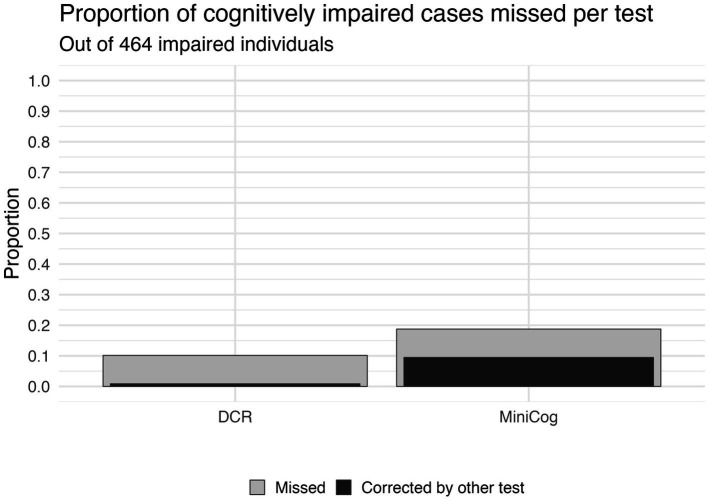
The sensitivity of each scoring approach based on a score cutoff of 4. The gray bars depict the proportion of cognitively impaired participants that were missed by each test. The black bars within the gray bars show the proportion of those missed participants that would have been correctly classified by the opposite test. Not only did the DCR have fewer misses (10.1% vs. 18.7% for Mini-Cog), but it would also correct a higher percentage of cases missed by the Mini-Cog (50.5%) than vice versa (8.5%). Further, 3 of the 47 missed by DCR are DLAD (8%), compared to 18 of the 87 missed by the Mini-Cog being DLAD (20%).

**Figure 4:**
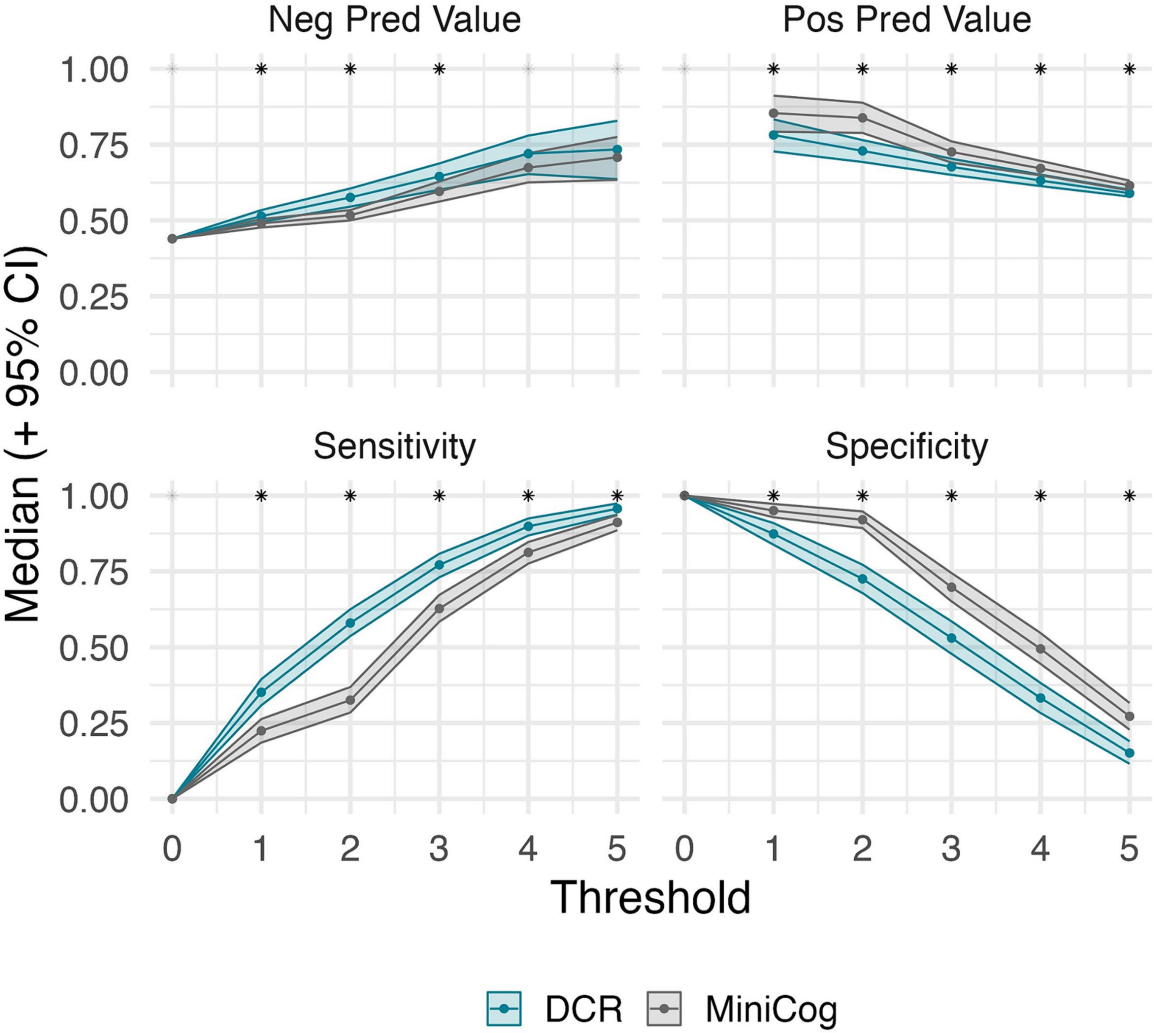
Threshold-wise classification performance for DCR and Mini-Cog, and their bootstrap 95% CI (where scores equal or greater than the threshold are considered unimpaired). Asterisks denote the significance of their difference (estimated at an alpha of 0.05). DCR maintains significantly higher sensitivity regardless of threshold. A significant difference was met if the bootstrap 95% CI of the differences between tests did not include zero. The lack of values for positive predictive value at 0 is due to neither test predicting impaired cases at that threshold. This shows that the DCR is more sensitive than Mini-Cog regardless of threshold. A tendency for greater negative predictive values suggests that a higher percentage of the individuals categorized as unimpaired by the DCR are indeed so.

Figure 2 shows the number of individuals in each diagnostic group at each score level for DCR vs. Mini-Cog rules. DCR classified more unimpaired individuals as impaired (false positives) with scores below 3 (*N* = 171) than did Mini-Cog (*N* = 110), but DCR also captured more true positives (*N* = 357) than Mini-Cog (*N* = 291). Figure 3 shows that, out of 464 total CI participants, when using a more lenient cutoff score of 4 (see Methods for rationale), DCR scoring was significantly more accurate in finding *a priori* CI participants (missed *N* = 47, or 10.1%; *N* MCI = 44; *N* DLAD = 3) (*χ*^2^ = 13.26, *p* < 0.0005) than Mini-Cog scoring (missed *N* = 87, or 18.7%; *N* MCI = 69; *N* DLAD = 18). Finally, Figure 4 shows that DCR was significantly more sensitive than the Mini-Cog regardless of threshold (all 95% CI of differences over 0), though at the expense of specificity. However, the tendency toward higher NPV shows that a higher percentage of the individuals categorized as unimpaired by the DCR are indeed so.

## Discussion

Our results build on existing evidence about the performance of the DCR, a next-generation version of the Mini-Cog, in detecting cognitive impairment in a well characterized sample of clinical trial participants. Although there was general agreement between the two blinded Raters of the Mini-Cog, our results demonstrate that even highly experienced neuropsychologists may differ in manual scoring leading to incorrect classification in some cases. Furthermore, while correlations between the Mini-Cog and the DCR scores were strong, the DCR missed substantially fewer individuals with cognitive impairment, supporting its enhanced detection capabilities. This evidence further supports that the process-driven performance analysis in the clock drawing test captures clinically relevant information effectively and efficiently, optimal in clinical practice. Current evidence suggests that the DCR could be relatively free of bias arising from cultural and socio-economic factors, suggesting it may be suitable for screening underrepresented populations and at scale (Jannati et al., 2023b).

We report strong general Mini-Cog scoring agreement between highly trained clinicians. Still, variability in their ratings led to more missed cases than the DCR scoring. The Mini-Cog is arguably the simplest manually-scored cognitive screening tool to administer and score, which requires minimal training of allied healthcare professionals such as medical assistants, nurses, and advanced practice clinicians (nurse practitioners or physician assistants). However, scoring between raters—despite its simple scoring rules—can vary, and may affect the cognitive classification of individuals. On the other hand, the DCR requires minimal administrator training and provides reliable results across different testing environments since it is scored automatically. The DCR scoring algorithm incorporates insights from the process of task performance—much the way an experienced clinician would do—not only from the final output. This process is referred to as the *Boston Process Approach* to neuropsychological assessment (Libon et al., 2022).

Recent studies have shown that clock drawing features, such as the shape and size of the clock, are associated with dementia (Davis et al., 2014; Bandyopadhyay et al., 2022). People with dementia have also been shown to be more stimulus-bound in the clock drawing process (Pittock et al., 2023), a feature that can be only partially observed from the final product alone. The DCR benefits from added context by assessing cognitive performance on two conditions: Copy Clock and Command Clock. Command Clock requires intact attention, auditory comprehension (receptive language), semantic memory, executive function, and visuoconstructional abilities, whereas Copy Clock relies primarily on visuospatial, attention, and executive function skills (Libon et al., 1993; Shulman, 2000; Cosentino et al., 2004; Yuan et al., 2021). This process-based approach provides the additional benefit of enabling the differentiation of MCI subtypes (Matusz et al., 2022).

The moderate correlation between the DCR and Mini-Cog scores should not be interpreted to indicate that these two assessments are equivalent. Rather, our results suggest that the strength of the correlation was impacted by the fact that the DCR is more sensitive for detecting impairment than the Mini-Cog; the DCR reduced the number of missed cases by the Mini-Cog by half (87 to 47). While the DCR and manually scored Mini-Cog take about the same time to administer [less than 3 min (Borson et al., 2000; Borson et al., 2003)], the DCR provides process-based analysis of multiple cognitive domains and immediate results that can be uploaded to the patient’s electronic health record (EHR).

It is important to emphasize differences in scoring for the Mini-Cog (a paper-and-pencil test) and DCR assessments (a process-driven score). Raters received specific instructions to apply the original Mini-Cog scoring criteria to the final clock images captured by the DCR. The clock face circle is typically provided for patients in the Mini-Cog and does not contribute to the scoring. The DCR, however, requires a circular clock face to be drawn from memory following verbal instructions by the person completing the test, thereby enabling assessment of semantic memory and visuoconstructional skills, neurocognitive functions that are not assessed to the same extent by the task design in the traditional Mini-Cog. Further, the drawing of the clock face, which is not completed in the paper-and-pencil Mini-Cog, is an important contributor to the DCR score and shows clinical significance in detecting cognitive impairment (Davis et al., 2014; Rentz et al., 2021; Bandyopadhyay et al., 2022). Finally, the Mini-Cog clock instructions allow administrators to “Repeat instructions as needed, as [it] is not a memory test.” In contrast, the DCR requires individuals to recall the test instructions, which on its own can be considered a low-load memory test.

The nuances of the process-driven score increase sensitivity when identifying cognitive impairment. For example, a drawn clock that correctly includes all 12 numbers and time would receive full credit on the Mini-Cog. However, the DCR scoring takes into account the spacing and placement of the clock components (i.e., drawing process, number, length, and variability of pauses in drawing, and planning). Additionally, one can lose all points on the Mini-Cog clock scoring for incorrect hand placement, whereas the DCR takes into account hand placement as one piece of the scoring algorithm. Further, it helps to elucidate the source of the added sensitivity of the DCR in general cognitive screening.

A limitation of the present study is that, by design, the study sample comprised individuals who had been independently classified as cognitively normal, mildly impaired, or having dementia likely due to AD in approximately equal numbers, all of whom were enrolled in a clinical trial focused on AD. In addition, we combined the two cognitively impaired groups into a single group for analysis, such that only one third of the sample had been classified as cognitively normal. This distribution may have inflated the performance of both Mini-Cog and DCR over what would be observed in an epidemiological sample with lower proportions of impaired individuals and a wider range of potential confounds from sociodemographic and medical conditions. Our results should therefore be interpreted in that context. To further the generalizability of these results, tests of the DCR’s performance in more heterogeneous groups, such as those seen in diverse health care settings, are needed to substantiate its use for detection of undiagnosed patients where specificity may be more applicable than sensitivity.

## Conclusion

This research supports the value of the DCR as a sensitive, automated process-driven digital cognitive assessment, and a next-generation evolution of the Mini-Cog. The DCR enables the capture of neuropsychological behavior helpful in detecting emergent cognitive impairment not attainable with traditional analog versions of these tests. The added value of the DCR over the Mini-Cog was observed in higher sensitivity for detection of cognitive impairment, further enhancing the ability of primary care providers to take action to help their patients with mild cognitive impairment previously not detected.

## Data Availability

The raw data supporting the conclusions of this article will be made available by the authors, without undue reservation.

## Footnotes

^1^ https://globalalzplatform.org/

^2^ https://mini-cog.com/scoring-the-mini-cog/
